# Trends in the population knowledge, attitudes and practices toward COVID-19 in the Buea municipality two months after the onset of the pandemic in Cameroon

**DOI:** 10.11604/pamj.2020.37.134.24821

**Published:** 2020-10-07

**Authors:** Forlemu Vanessa Mandaah, Tendongfor Nicholas, Seraphine Nkie Esemu, Amana Bokagne Therese Vanessa, Kouam Talla Gilchrist Destin, Nganyewo Cynthia Atiepoh, Lambou Fopa Vanessa

**Affiliations:** 1 Department of Microbiology and Parasitology, University of Buea, Buea, Cameroon,; 2 Department of Public Health and Hygiene, University of Buea, Buea, Cameroon,; 3 Laboratory for Emerging Infectious Diseases, University of Buea, Buea, Cameroon,; 4 Department of Biochemistry and Molecular Biology, University of Buea, Buea, Cameroon

**Keywords:** COVID-19 pandemic, trend, knowledge, attitude, practices, Buea municipality

## Abstract

**Introduction:**

two months into the COVID-19 pandemic in Cameroon, we assess the trend in the knowledge, attitude and practices of the population with the progression of the disease and the implementation of preventive methods put in place by the government of Cameroon and health partners organizations in response to the pandemic.

**Methods:**

this was a cross-sectional study conducted in selected health areas in the Buea municipality. A questionnaire was administered at the onset and two months later to collect data on the participants´ knowledge, attitude and practices towards COVID-19. The data were analyzed in SPSS version 25.

**Results:**

a total of 480 and 680 participants were sampled at onset and two months later respectively. Of the 26 indicators of knowledge assessed, 22 (84.61%) showed significant increase (p<0.05) with the most significant changes observed with the symptoms, mode of transmission and prevention of the disease. The overall proportion of people with correct knowledge moved from 9.1% at onset to 41.4% two months after. Only 1.5% of participants had poor knowledge of the pandemic two months after against 14.2% at the onset. There was a significant (p<0.05) positive change in the population perception, attitude and practices toward COVID-19 two months after the onset of the pandemic.

**Conclusion:**

the population knowledge, attitude and practices on COVID-19 showed a positive trend two months after the onset of the pandemic. The implementation of government and health stakeholder preventive measures together with the incidence of the pandemic in Cameroon probably had a positive impact on the positive trend observed. There is a need for continuous sensitization to completely fill the knowledge gap of the population on COVID-19.

## Introduction

The world is experiencing, since December 2019, the COVID-19 pandemic which has claimed several thousand lives worldwide. COVID-19 emerged in Wuhan city, the capital of China's Hubei province and has since spread globally, resulting in the ongoing coronavirus pandemic [1]. It was declared as a global pandemic by WHO on 12^th^ March 2020 [2]. The first case was reported in Cameroon on March 6^th^, 2020 [3]. As of the third week of June 2020, more than 11,000 cases had been reported with more than 300 deaths [4]. In response to the COVID-19 pandemic in Cameroon, the government and health partners put in place a series of measures to prevent the transmission of the pandemic. Among these measures were the closure of borders and schools, prohibition of gatherings of more than fifty people, respect of social distancing, postponement of school and university competitions, closure of bars, restaurants and entertainment spots from 6 P.M., a toll-free number 1510 set up for the mobilization of rescue teams and quarantine [5]. By April 9^th^ 2020, additional measures put in place included compulsory wearing of masks in public places, confinement imposed on persons at risk and intensification of educational efforts through audio-visual and print media.

A study conducted in Buea municipality at the onset of the pandemic in Cameroon showed a low level of knowledge of the population of the COVID-19 pandemic [6]. As a result of the progression of the pandemic in Cameroon and the population resistance to comply with government and stakeholder preventive measures, the government reinforced the implementation of preventive measures by putting fines on defaulters, making provision for hand washing points in public places and distribution of soap and hand sanitizers to the population. At the time the baseline study [6] was conducted, they were 10 reported cases of corona virus in Cameroon with no case in the South West region. Two months later, more than 11,000 cases were reported with the disease present in all the 10 regions of Cameroon [4]. This study aimed at assessing the trend of the population knowledge, attitude and practice of the COVID-19 pandemic in the Buea municipality.

## Methods

**Study site:** the study was carried out in Buea, the administrative head quarter of the South West region of Cameroon, one of the regions affected by the Anglophone crisis since 2016. The total population of Buea as estimated by the health population denominators (2013) was 300,000 inhabitants [7]. Many internally displaced people from neighbouring villages affected by the Anglophone crisis since 2016 have found refuge in Buea municipality. The epidemiological data were collected in communities selected in five health areas (Buea road, Molyko, Muea, Bokwango and Tole) out of the eight health areas in the Buea health district. The choice of these health areas for sampling was motivated by accessibility, security and population density.

**Study design and population:** the study was a cross-sectional and comparative study involving two independent samples in which a questionnaire was administered to assess the trend in participants´ knowledge of COVID-19 two months after the onset of the pandemic in Cameroon. Data were collected at the beginning (March 2020) and two months later (May 2020) into the pandemic and the results were discussed with respect to the government response measures and the progression of the pandemic in Cameroon. The accessible population was made up of males and females, aged 18 years or older and residing in the selected health areas in Buea municipality. Study participants were recruited from households and hot spots (population gathering points) and all of them were interviewed after providing a written informed consent. A minimum sample size of 384 was determined using the Lorenz formula with an expected proportion of the population having accurate knowledge of the virus adopted from a study conducted on a related viral infection (SARS) [8].

**Sample collection tools:** the sample collection tool used in the study was described in our previous study [6] adapted from the WHO guidelines on clinical manifestation and prevention of the COVID-19 [9]. The same tool was used to collect data at the onset of the pandemic and two months into the pandemic in Cameroon.

**Sampling technique:** this study was a two-stage clustered (health area and community based) cross-sectional questionnaire-based survey conducted in five health areas purposefully selected based on accessibility, security and population density. In each health area, two to five communities were randomly selected and data were collected from households and hot spots (meeting points, motor parks, restaurants and workshops). In each household visited, at most two persons were interviewed. Care was taken to include in the sample individuals of different age groups, gender, education and profession. The questionnaire was administered to participants in English language. Participants who were literate were allowed to fill the questionnaire themselves, whereas for those who could not read or write, the questionnaire was administered in “pidgin English”, a local language commonly spoken and understood in the area.

**Data analysis:** the data collected were entered into a template created in Epi Info version 7.2 and analyzed in the Statistical Package for the Social Sciences (SPSS) version 25 (SPSS, Inc., Chicago, IL, USA). Indicators used to evaluate the trend were awareness, knowledge of the disease, transmission, clinical manifestations and mode of prevention, population attitude and practices as previously described [6]. A comparison between the knowledge level, attitude and practices at the onset and two months into the epidemic was done using the Chi-square test of independence. A p-value<0.05 was considered statistically significant.

**Ethical considerations:** ethical clearance (2020/1203-04/UB/SG/IRB/FHS) was obtained from the Institutional Review Board of the Faculty of Health Sciences, University of Buea. An administrative clearance was gotten from the regional delegation of public health, South West region. The various participants gave their consent before being enrolled into the study. For participants less than 21 years, an assent was obtained from the participant in addition to the consent obtained from the parents or guardians.

## Results

**Socio-demographic characteristics of the study participants:** a total of 480 and 680 participants were interviewed at the beginning and two months following the onset of the pandemic respectively. With the exception of marital status and profession that showed a significant difference (p<0.05) in the distribution of the two samples, there was no significant difference in the other demographic characteristics of the two samples. Most participants were males, single, christians, with tertiary level of education. The age group 20 - 29 years was most represented (Table 1).

**Table 1:** socio-demographic characteristics of the study participants

Characteristic		Onset	Two months after	p-value
Health area	Bokwango	90 (7.8)	130 (11.2)	
	Buea Road	120 (10.3)	186 (16.0)	
	Molyko	110 (9.5)	195 (16.8)	0.170
	Muea	100 (8.6)	98 (8.4)	
	Tole	60 (5.2)	71 (6.1)	
Age group (years)	<20	49 (4.2)	70 (6.0)	
	20 - 29	237 (20.7)	335 (28.9)	0.917
	30 - 39	114 (9.8)	168 (14.5)	
	40 - 49	47 (4.1)	67 (5.8)	
	50 - 59	17 (1.5)	25 (2.2)	
	60+	16 (1.4)	15 (1.3)	
Gender	Female	211 (18.2)	309 (26.6)	0.617
	Male	269 (23.2)	371 (32.0)	
Marital status	Co-habiting	22 (1.9)	13 (1.1)	0.037
	Divorced	6 (0.5)	6 (0.5)	
	Married	167 (14.4)	223 (19.2)	
	Single	285 (24.6)	438 (37.8)	
Education	None	25 (2.2)	31 (2.7)	0.814
	Primary	23 (2.0)	37 (3.2)	
	Secondary	165 (14.2)	221 (19.1)	
	Tertiary	267 (23.0)	391 (33.7)	
Religion	Christian	445 (38.4)	631 (54.4)	0.670
	Muslim	21 (1.8)	34 (2.9)	
	Pagan	14 (1.2)	15 (1.3)	
Household size	1	48 (4.1)	55 (4.7)	0.168
	2 - 3	132 (11.4)	204 (17.6)	
	4 - 6	198 (17.1)	250 (21.6)	
	>6	102 (8.8)	171 (14.7)	
Profession	Employed	256 (22.1)	276 (23.8)	0.000
	Student	198 (17.1)	277 (23.9)	
	Unemployed	26 (2.2)	127 (10.9)	

**Figure 1:**
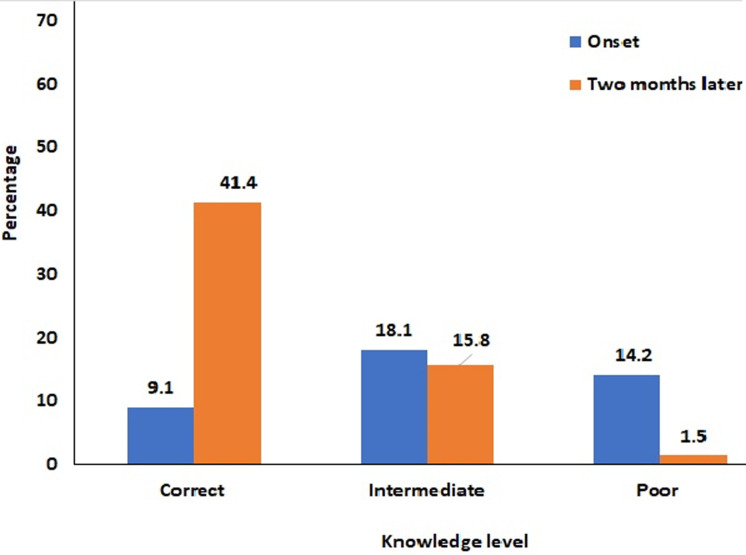
trend in participants knowledge of COVID-19 pandemic (χ2= 333.16, p=0.000)

**Trend in the participants level of knowledge of COVID-19:** overall, there was a significant (p<0.05) increase in participants knowledge level of COVID-19 from the onset to two months into the pandemic. The proportion of people with good knowledge moved from 9.1% at onset to 41.4%. Only 1.5 % participants had poor knowledge two months into the pandemic compare to 14.2 % at the onset (Figure 1). Table 2 shows the shift in the participants´ knowledge of COVID-19 two months after the onset of the pandemic. Of the 26 indicators assessed, 22 (84.61%) showed significant increase in knowledge, 3 (11.53%) a significant decreased and 1 (3.87%) did not change with time. For the indicators of awareness, the knowledge of the causative agent did not vary significantly (p=0.63) after two months. There was a significant (p=0.006) decrease in awareness of the reservoir. All the other indicators of awareness increased significantly (p<0.05) two months after the onset of the pandemic. There was a significant (p<0.005) increase of participants knowledge of the symptoms of the COVID-19 two months into the pandemic. The most significant increase was observed with fever (53.1% to 86.6%), shortness of breath (32.7% to 76.6%) and runny nose (36.5% to 76.8%).

**Table 2:** trend in participants´ knowledge of COVID-19 in the Buea municipality

Indicators		Onset	Two months after	p-value
Awareness of COVID-19	Name of the disease	443 (92.3)	651 (95.7)	0.013
	Reservoir	291 (60.6)	357 (52.5)	0.006
	Country of origin	443 (92.3)	652 (95.9)	0.009
	Other country affected	375 (78.1)	618 (90.9)	0.000
	Causative agent	370 (77.1)	516 (75.9)	0.635
	Cases in Cameroon	305 (63.5)	470 (69.1)	0.047
	First case in Cameroon	274 (57.1)	470 (69.1)	0.013
Site of infection	Respiratory tract	394 (82.1)	589 (86.6)	0.034
Symptoms of the infection	Fever	255 (53.1)	579 (85.1)	0.000
	Cough	367 (76.5)	592 (87.1)	0.000
	Sore throat	167 (34.8)	284 (41.8)	0.016
	Runny nose	175 (36.5)	522 (76.8)	0.000
	Shortness of breath	157 (32.7)	521 (76.6)	0.000
Mode of transmission	Contact with infected persons	358 (74.6)	439 (64.6)	0.000
	Contact with infected objects	217 (45.2)	448 (65.9)	0.000
	Respiratory droplets	110 (22.9)	185 (27.2)	0.980
	Particles sneezed or coughed out	249 (51.9)	502 (73.8)	0.000
Mode of prevention	Avoid close contact with patients	394 (82.1)	530 (77.9)	0.084
	Avoid touching eyes, nose and mouth	209 (43.5)	519 (76.3)	0.000
	Stay home	192 (40.0)	391 (57.5)	0.000
	Cover cough	206 (42.9)	424 (62.4)	0.000
	Regular hand washing	284 (59.2)	554 (81.5)	0.000
	Cough and sneeze in the elbow	99 (20.6)	432 (63.5)	0.000
	Greet people without shaking hands	221 (46.0)	424 (62.4)	0.000
	Social distancing	152 (31.7)	392 (57.6)	0.000
	Wearing of face mask	268 (55.8)	566 (83.2)	0.000

For the mode of transmission, there was no significant change in knowledge on transmission through respiratory droplets. A decrease in knowledge with respect to contact with infected persons was observed but it was not significant. The only significant increase in knowledge was observed with transmission through contact with infected objects (45.2% to 65.9%). For the mode of prevention, with the exception of prevention by avoiding contact with infected persons that did not change significantly, all the other indicators showed significant (p<0.05) increase in the knowledge of the mode of prevention two months after the onset of the pandemic.

**Trend in the practices of the population with respect to COVID-19:** with respect to the practices of the population, there was a significant improvement in the knowledge of face mask and hand sanitizer. Acquisition of face mask and hand sanitizer by participants also showed a significant increase (p<0.05). The most significant increase was observed with purchase of face masks (from 0.9 % to 56.1%) (Table 3).

**Table 3:** trend in the practices of population with respect to COVID-19

Variables	Levels	Onset	Two months after	p-value
Knowledge of face masks	No	31 (2.7)	13 (1.1)	
	Yes	449 (38.7)	667 (57.5)	0.000
Bought your face mask for personal use	No	376 (32.4)	29 (2.5)	
	Yes	104 (9.0)	651(56.1)	0.000
Knowledge of hand sanitizer	No	54 (4.7)	43 (3.7)	
	Yes	426 (36.7)	637 (54.9)	0.003
Bought a hand sanitizer for personal use	No	323 (27.8)	154 (13.3)	
	Yes	157 (13.5)	526 (45.3)	0.000

**Figure 2:**
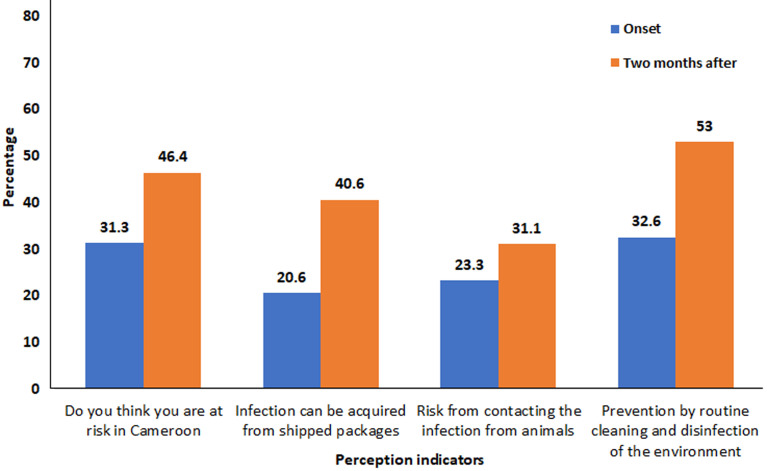
trend in the perception of the population with respect to COVID-19

**Trend in the perception of the population with respect to COVID-19:** the perception that infection could be acquired from shipped packages and can be prevented by routine cleaning of the environment also increased significantly (p=0.000) two months into the pandemic. Being at risk of infection in Cameroon and contacting it from animals around increased from onset to two months into the pandemic but the difference was not significant (Figure 2).

**Trend in the participant´s attitude to the COVID-19 pandemic:** a significant (p<0.001) improvement in the participants attitude with respect to the use of emergency number and going to the hospital in case of an infection was observed. The proportion of participants that reported they will resort to auto-medication in case of an infection with COVID-19 dropped significantly (p<0.001) two months after the onset of the pandemic (Table 4).

**Table 4:** attitude of participants to the COVID-19 pandemic

Variables	Levels	Onset	Two months after	p-value
Call emergency number	No	255 (22.0)	206 (17.8)	0.000
	Yes	225 (19.4)	474 (40.9)	
Meet native doctor	No	466 (40.2)	659 (56.8)	0.866
	Yes	14 (1.2)	21 (1.8)	
Auto medication	No	217 (20.0)	637 (58.7)	0.000
	Yes	189 (17.4)	43 (4.0)	
Meet a man of God	No	432 (37.2)	612 (52.8)	1.000
	Yes	48 (4.1)	68 (5.9)	
Go to the hospital	No	88 (7.6)	195 (16.8)	0.000
	Yes	392 (33.8)	485 (41.8)	

## Discussion

Given the serious threats posed by COVID-19 and the absence of a vaccine, implementation of preventive measures plays an essential role in reducing the infection rates and curbing the spread of the disease [10]. A good knowledge, attitudes and practices of these preventive measures by the population is critical in the successful control and prevention of the infection. This study assessed the change in the population knowledge, attitudes and practices of the COVID-19 pandemic with the progression of the outbreak in Cameroon and the implementation of government response measures to control and prevent the spread of the pandemic.

**Shift in participants´ knowledge of COVID-19:** the first case of COVID-19 was registered in Cameroon in early March 2020 [3]. We observed an increase in participants´ knowledge of COVID-19 two months after the onset of the pandemic. Of the 26 indicators of knowledge assessed, 19 (73.1%) showed significant increase in knowledge two months after the onset of the pandemic, indicating that the population has gained significant knowledge with the progression of the pandemic. All the indicators of symptoms and prevention showed significant increase indicating an increase awareness of the population of the symptoms and mode of prevention of the infection. This significant shift in knowledge could be justified in part by the prompt response and implementation of barrier and educational measures by the government of Cameroon and health stakeholders. In fact, since the beginning of the pandemic the government and professional health partners have not relented any effort in the education and sensitization of the population on the implementation of the barrier methods. Two months after the onset of the pandemic 41.4% had correct knowledge, indicating that more is still to be done in terms of population education on COVID-19 to completely fill the knowledge gap.

As a result of the progression of the pandemic characterized by a rising number of cases and deaths, people became more aware of the danger of the infection especially the mode of transmission and prevention. This contributed significantly to the reduction in the knowledge gap observed in the population of the Buea municipality at the onset of the pandemic. When the baseline data was collected in March 2020 only 10 cases of COVID-19 were reported in Cameroon and the South West region, where this study was carried out, had no COVID-19 cases yet. This could explain the knowledge gap observed in the population at the onset of the pandemic [6]. Two months after the onset of the pandemic in Cameroon, all regions have been affected including the South West region. A general increase was observed in the level of awareness of the population towards the disease; like knowing the name of the disease, towns affected in Cameroon, origin of the disease, causative agent and reservoir. Data from the Ministry of Public Health show a rise in the number of cases and death since the onset of the pandemic (MOH or WHO statistics on trends in COVID-19 in Cameroon). The constant increase in the number of cases and deaths due to COVID-19 in the country could be a contributing factor to the population awareness and knowledge gain.

**Participants´ practices and attitudes toward COVID-19:** the practices of the participants with respect to COVID-19 also showed some improvement two months after the onset of the pandemic. A high proportion of the participants had good practices towards implementing the preventive measures. The proportion of participants who had acquired face masks and hand sanitizers for their protection was significantly higher compared to the proportion at the onset of the pandemic. This was a good indication towards an effective prevention of the disease. This great improvement could be due to the reinforcement of the implementation of preventive measures as well as the progression of the pandemic, which is similar to a study done in China on measures put in place by the government to fight COVID-19 [11]. As a result of the reluctancy of the population to comply with the government measures, there was a reinforcement with an introduction of fines on defaulters. This certainly had a positive impact on the population practices. Generally, there was a positive change in the population attitude toward COVID-19 with the progression of the disease. They were more knowledgeable about reporting to the hospital and calling the emergency number in case of an infection as well as not resorting to auto medication.

Despite the considerable change in knowledge and awareness, positive change in the population attitude and practice, the number of cases of COVID-19 has not stopped rising in the Buea municipality in particular and Cameroon in general. In a space of three months after the onset, Cameroon has registered more than 12,000 cases with more than 300 deaths. This is an indication that the pandemic continues to spread despite significant change in knowledge, attitude and practice of the population. In this study even though we observed significant change in the knowledge, attitude and practice, there is still an urgent need to continue sensitization and strengthening the implementation of barriers measures put in place by the government to slow down this pandemic. The relaxation of these measure may exacerbate the crisis. One of the limitations of the study is that a qualitative arm was not included and the participants who took part in the study at the onset were not necessarily those who took part in the survey two months after.

## Conclusion

The Buea population knowledge on COVID-19, attitudes and practices improved significantly two months after the onset of the COVID-19 pandemic in Cameroon. The implementation of government and health stakeholder preventive measures together with the incidence of the pandemic in Cameroon probably had a positive impact on the positive trend observed in the Buea population. There is a need for continuous sensitization to completely fill the knowledge gap of the population on COVID-19.

### What is known about this topic

- The first cases of COVID-19 were declared in Cameroon on the 6^th^ of March 2020;
- The government of Cameroon in response to the COVID-19 pandemic put in place a series of measures to prevent the spread of the disease in Cameroon;
- The knowledge level, attitude and practices, of the population toward COVID-19 at the onset of the pandemic was poor.

### What this study adds

- The knowledge gap of the population of Buea toward COVID-19 has reduced significantly two months post onset of the pandemic;
- There was a positive change in the participants´ attitudes and practices toward COVID-19 two months after the onset of the pandemic;
- All indicators of prevention and transmission assessed showed a significant increase with the progression of the pandemic.
